# Supplementary material for: Unified mass imaging maps the lipidome of vertebrate development
Source: Nat Methods. 2025 Sep 3;22(9):1981–94. doi: 10.1038/s41592-025-02771-7 (PMC12446072; doi:10.1038/s41592-025-02771-7)
Supplement: Supplementary file 1 — Supplementary Figs. 1–7. [file 41592_2025_2771_MOESM1_ESM.pdf]

nature methods

Supplementary information

https://doi.org/10.1038/s41592-025-02771-7

Unified mass imaging maps the lipidome of vertebrate development

In the format provided by the authors and unedited

Unified Mass Imaging Maps the Lipidome of Vertebrate Development
Supplementary Information

Halima Hannah Schede[*, 1,2], Leila Haj Abdullah Alieh[*, 1,2], Laurel Ann Rohde[1], Antonio Herrera[2], Anjalie Schlaeppi[3], Guillaume Valentin[4], Alireza Gargoori Motlagh[2], Albert Dominguez Mantes[1,2], Chloe Jollivet[1], Jonathan Paz-Montoya[1], Laura Capolupo[1,5], Irina Khven[2], Andrew C. Oates[1], Giovanni D'Angelo[**, 1], and Gioele La Manno[**, 2]

[1]Institute of Bioengineering, School of Life Sciences, Swiss Federal Institute of Technology (EPFL), Lausanne, Switzerland
[2]Brain Mind Institute, School of Life Sciences, Swiss Federal Institute of Technology (EPFL), Lausanne, Switzerland
[3]Bioimaging and Optics Core Facility, School of Life Sciences, Swiss Federal Institute of Technology (EPFL), Lausanne, Switzerland
[4]Center of PhenoGenomics, School of Life Sciences, Swiss Federal Institute of Technology (EPFL), Lausanne, Switzerland
[5]Friedrich Miescher Institute for Biomedical Research, Basel, Switzerland
[*]These authors contributed equally
[**]These authors contributed equally to the supervision of the project

1 Supplementary Figures

This document contains all Supplementary Figures for the article.

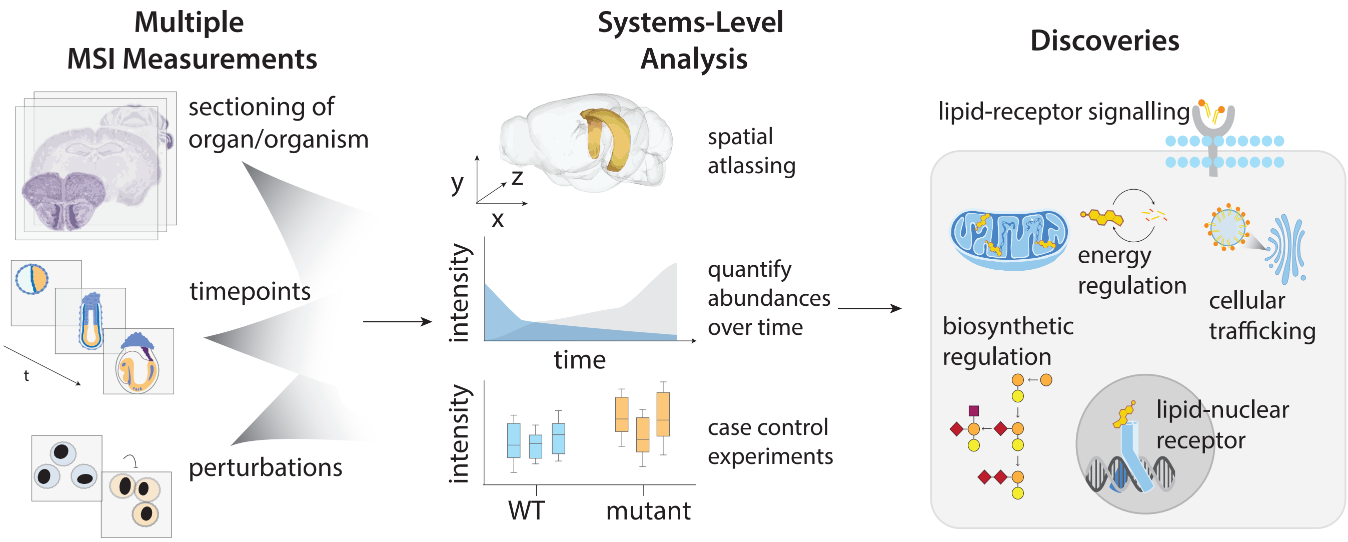

Supplementary Figure 1: **Workflow: from multiple MSI acquisitions to discovery using the uMAIA analysis framework.** General workflow enabled by uMAIA. From the left to the right: different MSI datasets consisting of collections of sections across organs, time points, or perturbations; aggregation and homogenization of the acquisitions to perform a system-level analysis; discovery of processes regulated region-specifically by lipids and metabolites.

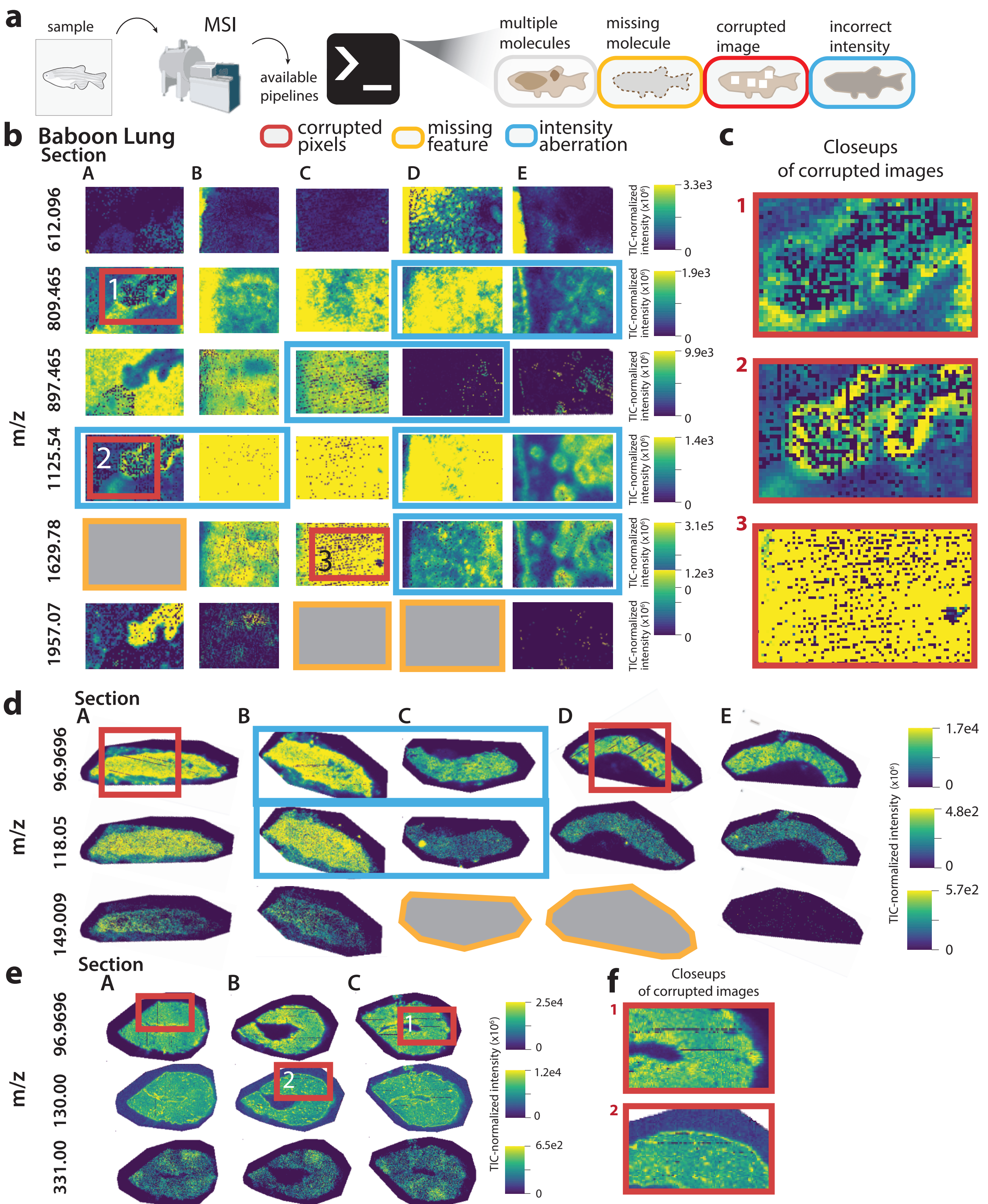

Supplementary Figure 2: **Showcasing different artifacts and data limitations impairing the analysis of MSI datasets (a)** Schematic illustrating the fact that MALDI-MSI data pipelines can produce outputs with artifacts and inconsistencies that can impair the downstream analyses of a combined dataset. **(b)** Represenatative MSI images of metabolites (m/z in bold) from public MSI datasets of the Baboon lung (METASPACE, PNNL dataset). Images were extracted from METASPACE. Different kinds of artifacts and limitations of the data that impact downstream analyses are highlighted in different colors. **(c)** Close-ups of MALDI acquisitions in (b) showing a scattered-like pattern of dropouts corrupting MSI images. **(d)** Representative MSI images from the Human Kidney (METASPACE, NIH KMPM dataset). **(e)** Another set of MSI images from the Human Kidney. **(f)** Close-ups of the corrupted MALDI images in (e).

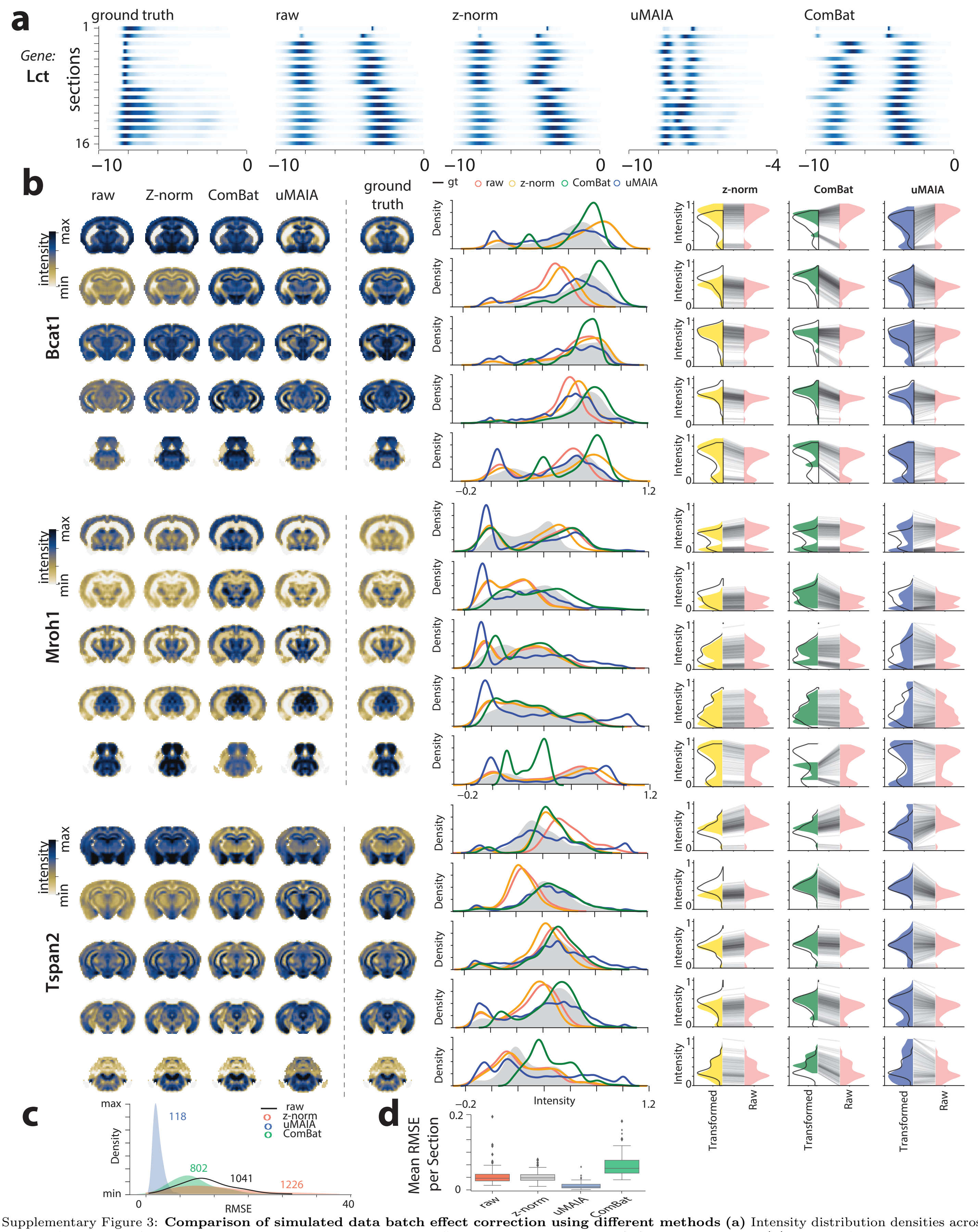

Supplementary Figure 3: **Comparison of simulated data batch effect correction using different methods (a)** Intensity distribution densities across sections for gene *Lct* for ground truth, simulated raw, z-normalized, ComBat-normalized and uMAIA-normalized data. **(b)** Images of genes *Bcat1*, *Mroh1*, *Tspan2* in ground truth, simulated raw, Z-normalized, Combat-normalized and uMAIA-normalized data (left) with corresponding intensity distributions (middle). Density plots of data corrected by the respective methods, with direct comparison to simulated raw data (pink distribution) where gray lines depict the intensity transformation and black solid line as the ground truth (right). **(c)** Density plots of RMSE scores for simulated raw data (black line) and data corrected with z-normalization, uMAIA, and ComBat. The number displayed above distributions in affiliated color indicates total RMSE. **(d)** Box plots representing interquartile ranges and mean RMSE per section for simulated raw data and data corrected with z-normalization, uMAIA, and ComBat.

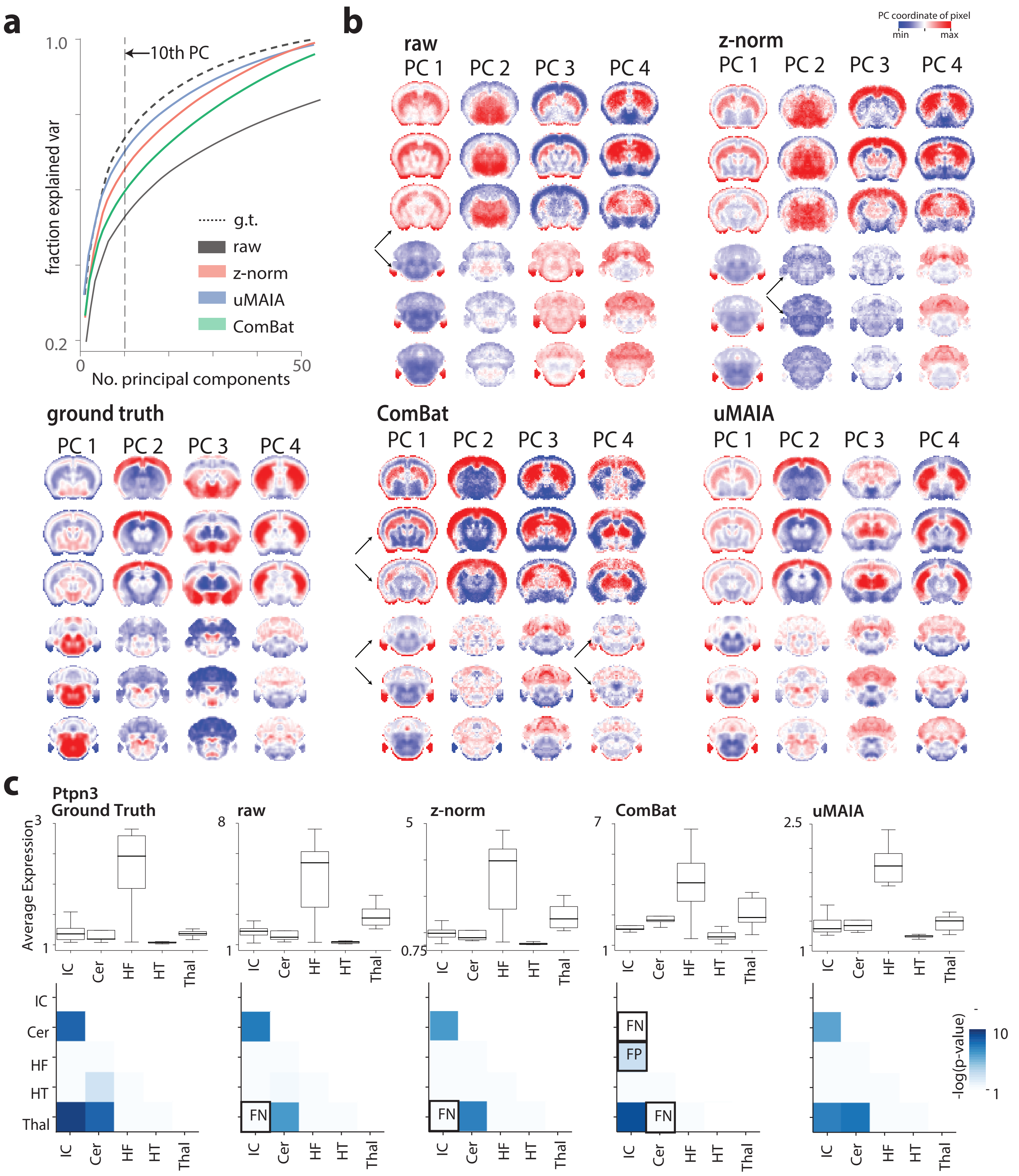

Supplementary Figure 4: **Evaluation of the rescue and preservation of ground truth signal after normalization using ABA-based MSI simulated data (a)** Line plot tracking the fraction of explained variance by different numbers of principal components for ground truth, simulated raw data, and normalization on the ABA-based simulation (see Methods). Dotted line indicates the 10th principal component. **(b)** Visualization of principal components for ground truth, simulated raw data and data corrected with z-normalization, ComBat and uMAIA. Black arrows indicate sections where significant batch effects still exist. **(c)** Average expression of gene *Ptpn3* in 5 major anatomical regions. Box plots indicate variability of expression across sections for each setting: ground truth, simulated raw data and data corrected with z-normalization, ComBat and uMAIA (above). Heatmaps display the -log(p-value) of t-tests between any pair of regions that can further be used to distinguish false negative and false positive detections with respect to ground truth (below).

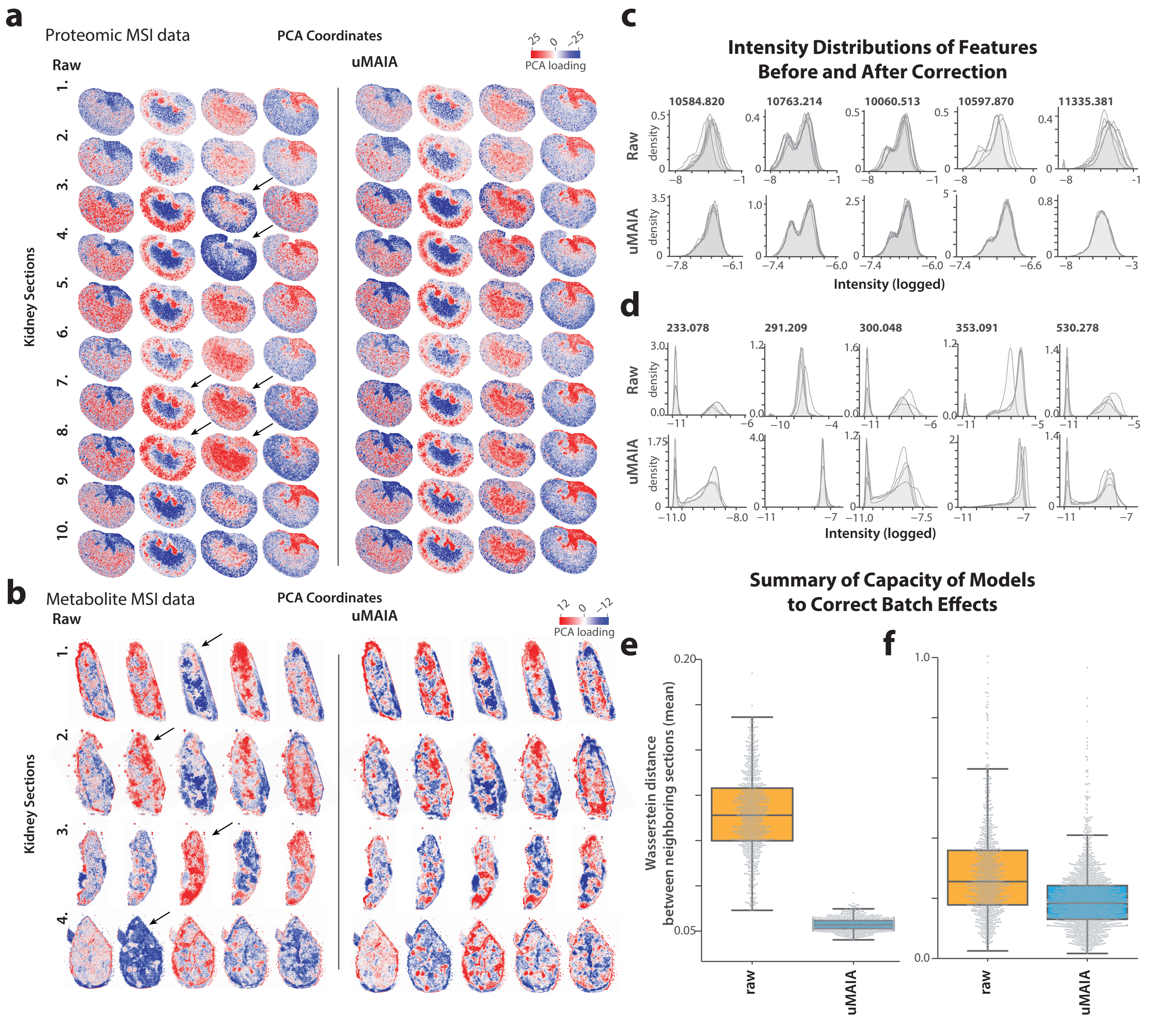

Supplementary Figure 5: **Performance evaluation of uMAIA on proteomic and metabolite MSI data (a)** Top 4 PC coordinates (columns) of 10 consecutive sections (rows) from kidney data (Oetjen et al., 2014) for raw data (left partition) and uMAIA-corrected data (right partition). Black arrows indicate sections where significant batch effects still exist. **(b)** Same as in (a), but with metabolite MSI data from the NIH kidney dataset. **(c)** Intensity distributions of compounds for all sections before (upper row) and after uMAIA correction (lower row) for proteomic MSI data. Bold number above panels indicate m/z of compound. **(d)** Same as in (c), but with metabolite MSI data from the NIH kidney dataset. **(e)** Average Wasserstein distance between neighboring sections across all compounds from the proteomic MSI dataset for raw (orange) and uMAIA-corrected (blue) data. **(f)** Same as in (e), but with metabolite MSI data from the NIH kidney dataset.

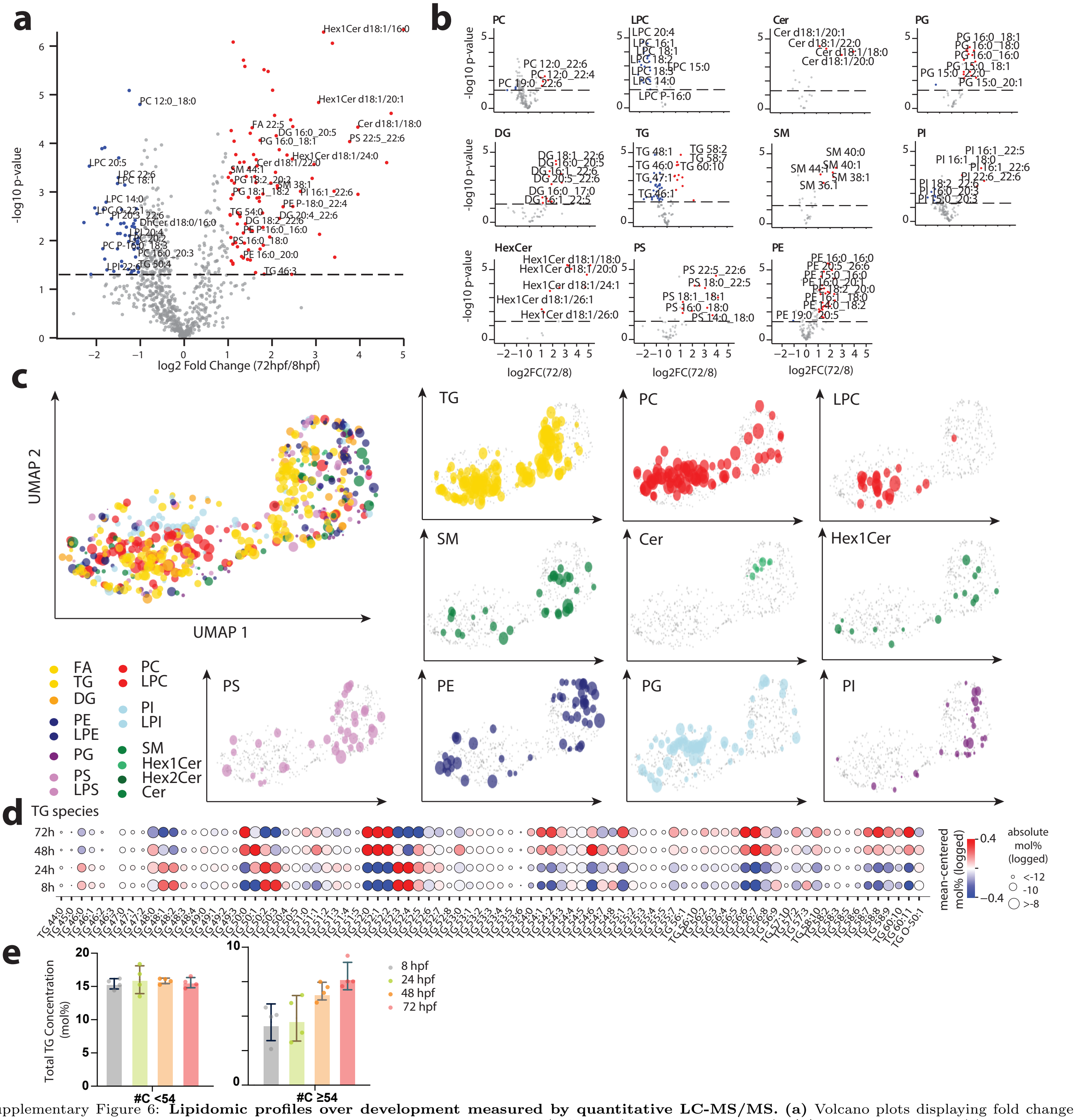

Supplementary Figure 6: **Lipidomic profiles over development measured by quantitative LC-MS/MS. (a)** Volcano plots displaying fold change between lipids detected in 72 hpf and 8 hpf zebrafish from quantitative LC-MS/MS data (Student's t-test, n=4). **(b)** Volcano plots as in (a) stratified by lipid class. **(c)** Low dimensional representation (UMAP) of lipids abundances measured by quantitative LC-MS/MS over developmental time color-coded by lipid class. Dot size is proportional to the log of measured concentration from LC-MS/MS for each lipid species. **(d)** Relative changes of TG species from bulk lipidomics across the time points. **(e)** Total TG concentrations over time points stratified by total carbon content. Error bar indicate standard deviation (n=4).

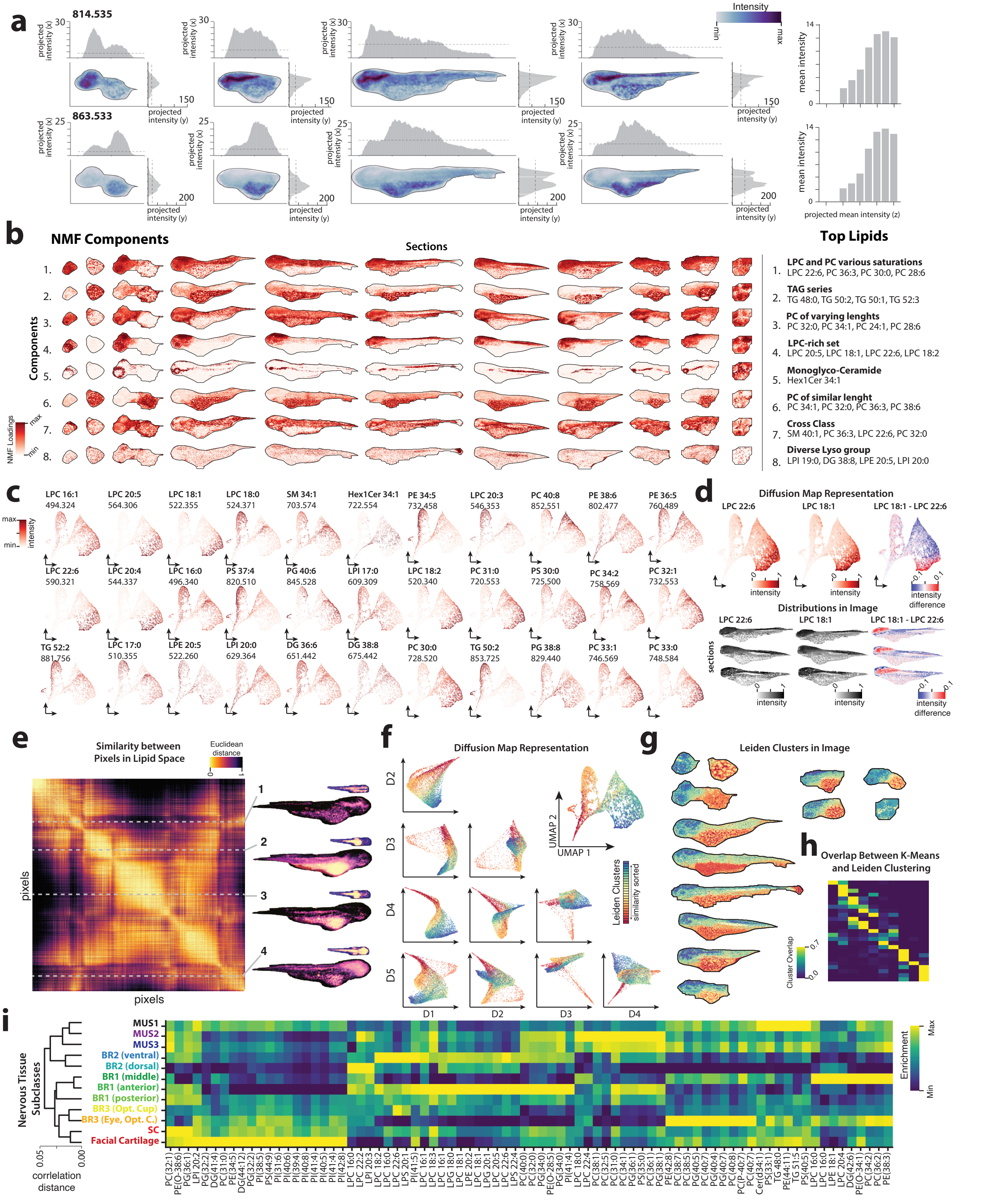

Supplementary Figure 7: **Lipid distributions and variability across 3 dimensions for the 72 hpf zebrafish embryo** **(a)** Visualization of the distribution of 2 lipids (rows, mz= 814.535, 863.533) in a set of four sagittal sections spanning the mediolateral axis. Histograms on the margins show the projected intensities across the x, y, and z axes. **(b)** Non-negative matrix factorization (NMF) of lipid distributions after feature selection and rescaling of features with corresponding Moran's I scores. Spatial distributions of the components are shown (left), with lipids representative of the component indicated (right). **(c)** Low-dimensional embedding of the zebrafish MSI pixels (UMAP of 5-component diffusion map representations) colored by lipid intensities. **(d)** The same scatter plots of two selected lipids LPC intensities and their differences (upper row) with corresponding images (lower row) **(e)** Heatmap of pairwise similarities between pixels lipid composition. The similarity metric used is Euclidean distance. On the right, the values of 4 rows of the matrix were visualized in correspondence with each pixel location to highlight the correspondence of the main blocks of the matrix with anatomical structures. **(f)** Scatter plot matrix of pairs of diffusion map axes, with corresponding 2D representation achieved using UMAP. **(g)** Visualization of Leiden clusters in image for different sections. **(h)** Confusion matrix of the Leiden and K-Means cluster assignments **(i)** Heatmap depicting lipid enrichment in different brain region clusters. All plots in the figure refer to the 72 hpf zebrafish embryo. Rows are sorted as indicated in the dendrogram on the right.
