# Supplementary material for: Unified mass imaging maps the lipidome of vertebrate development
Source: Nat Methods. 2025 Sep 3;22(9):1981–94. doi: 10.1038/s41592-025-02771-7 (PMC12446072; doi:10.1038/s41592-025-02771-7)
Supplement: Supplementary file 2 — Reporting Summary [file 41592_2025_2771_MOESM2_ESM.pdf]

nature portfolio

Corresponding author(s): G. La Manno, G. D'Angelo

Last updated by author(s): G. La Manno, G. D'Angelo

Reporting Summary

Nature Portfolio wishes to improve the reproducibility of the work that we publish. This form provides structure for consistency and transparency in reporting. For further information on Nature Portfolio policies, see our Editorial Policies and the Editorial Policy Checklist.

Please do not complete any field with "not applicable" or n/a. Refer to the help text for what text to use if an item is not relevant to your study.
For final submission: please carefully check your responses for accuracy; you will not be able to make changes later.

Statistics

For all statistical analyses, confirm that the following items are present in the figure legend, table legend, main text, or Methods section.

n/a	Confirmed	
☐	☑	The exact sample size (n) for each experimental group/condition, given as a discrete number and unit of measurement
☐	☑	A statement on whether measurements were taken from distinct samples or whether the same sample was measured repeatedly
☐	☑	The statistical test(s) used AND whether they are one- or two-sided<br>*Only common tests should be described solely by name; describe more complex techniques in the Methods section.*
☐	☑	A description of all covariates tested
☐	☑	A description of any assumptions or corrections, such as tests of normality and adjustment for multiple comparisons
☐	☑	A full description of the statistical parameters including central tendency (e.g. means) or other basic estimates (e.g. regression coefficient) AND variation (e.g. standard deviation) or associated estimates of uncertainty (e.g. confidence intervals)
☐	☑	For null hypothesis testing, the test statistic (e.g. F, t, r) with confidence intervals, effect sizes, degrees of freedom and P value noted<br>*Give P values as exact values whenever suitable.*
☐	☑	For Bayesian analysis, information on the choice of priors and Markov chain Monte Carlo settings
☐	☑	For hierarchical and complex designs, identification of the appropriate level for tests and full reporting of outcomes
☐	☑	Estimates of effect sizes (e.g. Cohen's d, Pearson's r), indicating how they were calculated

Our web collection on statistics for biologists contains articles on many of the points above.

Software and code

Policy information about availability of computer code

Data collection	MALDI MSI data were collected withTune from Thermofisher scientific. Described in the methods section.
Data analysis	Python 3.11, Fiji 2.9.0 and Ashlar .Source code at https://github.com/lamanno-epfl/uMAIA/tree/main. Described in methods section.

For manuscripts utilizing custom algorithms or software that are central to the research but not yet described in published literature, software must be made available to editors and reviewers. We strongly encourage code deposition in a community repository (e.g. GitHub). See the Nature Portfolio guidelines for submitting code & software for further information.

Data

Policy information about availability of data

All manuscripts must include a data availability statement. This statement should provide the following information, where applicable:

- Accession codes, unique identifiers, or web links for publicly available datasets
- A description of any restrictions on data availability
- For clinical datasets or third party data, please ensure that the statement adheres to our policy

The raw data can be retrieved on METASPACE in .IBD and .imzML format at https://metaspace2020.eu/project/uMAIA. HybISS data are available at the following link: https://zenodo.org/records/14170238 (native coordinates) and https://zenodo.org/records/14514399 (matching MSI).

Publicly available MALDI-MSI datasets were retrieved from METASPACE. Human kidney samples from the NIH Kidney Precision Medicine Project (ID: 2024-06-06_19h26m13s, 2024-06-06_19h22m09s, 2024-06-06_19h16m21s, 2024-06-06_19h08m24s, 2024-06-06_19h03m38s, 2024-06-06_18h57m58s, 2024-06-06_18h45m39s, 2024-06-06_18h40m58s)

Baboon lung samples from the Pacific Northwest National Laboratory (ID: 2024-06-11_18h30m14s, 2024-06-11_18h28m14s, 2024-06-11_18h35m41s, 2024-06-11_18h26m59s, 2024-06-11_18h43m25s)

Supplementary Table 1: Contains the set of all annotable m/z detections retrieved from MALDI-MSI data (in rows) with metadata in columns including, but not limited to, lipid quantities, potential isobars, sum compositions, specific lipid species identified from LC-MS/MS.

Supplementary Table 2: Contains all information retrieved from bulk LC-MS/MS quantification. These include 4 sheets: (1) lipid quantifications before normalization, (2) total protein content for each sample, (3) lipid quantifications after normalization, (4) a lipid transition table that lists lipid names with their precursor mz, retention time, ionisation and adduct.

Research involving human participants, their data, or biological material

Policy information about studies with human participants or human data. See also policy information about sex, gender (identity/presentation), and sexual orientation and race, ethnicity and racism.

Reporting on sex and gender	NA
Reporting on race, ethnicity, or other socially relevant groupings	NA
Population characteristics	NA
Recruitment	NA
Ethics oversight	NA

Note that full information on the approval of the study protocol must also be provided in the manuscript.

Field-specific reporting

Please select the one below that is the best fit for your research. If you are not sure, read the appropriate sections before making your selection.

☑ Life sciences ☐ Behavioural & social sciences ☐ Ecological, evolutionary & environmental sciences

For a reference copy of the document with all sections, see nature.com/documents/nr-reporting-summary-flat.pdf

Life sciences study design

All studies must disclose on these points even when the disclosure is negative.

Sample size	MSI dataset: two individuals per developmental stage were sampled; sample size was chosen to provide representative data while accounting for the labor-intensive nature of MSI technology; LC-MS/MS: four biological replicates (each consisting of 15 pooled individual embryos) were used per developmental stage; this number was chosen based on previously published protocol indicating that this sample size provides robust signal detection and reproducibility in LC-MS/MS. Morpholino experiments: at least six embryos were analyzed for each condition and experiment. This sample size is consistent with standards in developmental biology for detecting phenotypic effects.HybISS:only 2 embryos were used; for a qualitative assessement this sample size was sufficient
Data exclusions	Only normally developing wild-type embryos were used. Low-quality MSI data were excluded from the final dataset. Embryos that did not survive the morpholino injection were excluded. For HybISS, genes with few counts were not considered.
Replication	MSI atlases are based on one embryo densely sampled (every 20 um), with additional representative sections from a second embryo, for each developmental stage. Morpholino injections were repeated 3 times. HybISS was performed once on a sagittal section and once on a coronal section.All attempts at replication were succesful.
Randomization	No randomization was performed for the MSI data. For morpholinos experiments, different clutches were merged and random embryos groups were allocated to morpholinos or controls; a pool of non-injected siblings were kept at the same conditions to control for phenotypic defects not-related to the injections; no abnormalities were observed.
Blinding	For the morpholino experiment the phenotype was so strong that blinding was not deemed necessary.

Behavioural & social sciences study design

All studies must disclose on these points even when the disclosure is negative.

Study description	NA
Research sample	NA
Sampling strategy	NA
Data collection	NA
Timing	NA
Data exclusions	NA
Non-participation	NA
Randomization	NA

Ecological, evolutionary & environmental sciences study design

All studies must disclose on these points even when the disclosure is negative.

Study description	NA
Research sample	NA
Sampling strategy	NA
Data collection	NA
Timing and spatial scale	NA
Data exclusions	NA
Reproducibility	NA
Randomization	NA
Blinding	NA

Did the study involve field work? ☐ Yes ☐ No

Field work, collection and transport

Field conditions	NA
Location	NA
Access & import/export	NA
Disturbance	NA

Reporting for specific materials, systems and methods

We require information from authors about some types of materials, experimental systems and methods used in many studies. Here, indicate whether each material, system or method listed is relevant to your study. If you are not sure if a list item applies to your research, read the appropriate section before selecting a response.

Materials & experimental systems

n/a	Involved in the study	
☑	☐	Antibodies
☑	☐	Eukaryotic cell lines
☑	☐	Palaeontology and archaeology
☐	☑	Animals and other organisms
☑	☐	Clinical data
☑	☐	Dual use research of concern
☑	☐	Plants

Methods

n/a	Involved in the study	
☑	☐	ChIP-seq
☑	☐	Flow cytometry
☑	☐	MRI-based neuroimaging

Antibodies

Antibodies used	NA
Validation	NA

Eukaryotic cell lines

Policy information about cell lines and Sex and Gender in Research

Cell line source(s)	NA
Authentication	NA
Mycoplasma contamination	NA
Commonly misidentified lines (See ICLAC register)	NA

Palaeontology and Archaeology

Specimen provenance	NA
Specimen deposition	NA
Dating methods	NA

☐ Tick this box to confirm that the raw and calibrated dates are available in the paper or in Supplementary Information.

Ethics oversight	

Note that full information on the approval of the study protocol must also be provided in the manuscript.

Animals and other research organisms

Policy information about studies involving animals; ARRIVE guidelines recommended for reporting animal research, and Sex and Gender in Research

Laboratory animals	Danio rerio embryos up to 120 hours post-fertilization
Wild animals	No wild animals were used in the study
Reporting on sex	Danio rerio embryos at the stages considered in this study are considered female as sexual determination become apparent later on in development.
Field-collected samples	No field collected samples were used in the study
Ethics oversight	All zebrafish husbandry procedures have been approved and accredited by the federal food safety and veterinary office of the canton of Vaud (VD-H23)

Note that full information on the approval of the study protocol must also be provided in the manuscript.

Clinical data

Policy information about clinical studies
All manuscripts should comply with the ICMJE guidelines for publication of clinical research and a completed CONSORT checklist must be included with all submissions.

Clinical trial registration	NA
Study protocol	NA
Data collection	NA
Outcomes	NA

Dual use research of concern

Policy information about dual use research of concern

Hazards

Could the accidental, deliberate or reckless misuse of agents or technologies generated in the work, or the application of information presented in the manuscript, pose a threat to:

No	Yes	
✓	☐	Public health
✓	☐	National security
✓	☐	Crops and/or livestock
✓	☐	Ecosystems
✓	☐	Any other significant area

Experiments of concern

Does the work involve any of these experiments of concern:

No	Yes	
✓	☐	Demonstrate how to render a vaccine ineffective
✓	☐	Confer resistance to therapeutically useful antibiotics or antiviral agents
✓	☐	Enhance the virulence of a pathogen or render a nonpathogen virulent
✓	☐	Increase transmissibility of a pathogen
✓	☐	Alter the host range of a pathogen
✓	☐	Enable evasion of diagnostic/detection modalities
✓	☐	Enable the weaponization of a biological agent or toxin
✓	☐	Any other potentially harmful combination of experiments and agents

Plants

Seed stocks	NA
Novel plant genotypes	NA
Authentication	NA

ChIP-seq

Data deposition

☐ Confirm that both raw and final processed data have been deposited in a public database such as GEO.

☐ Confirm that you have deposited or provided access to graph files (e.g. BED files) for the called peaks.

Data access links<br>*May remain private before publication.*	NA
Files in database submission	NA
Genome browser session<br>(e.g. UCSC)	NA

Methodology

Replicates	NA
Sequencing depth	NA
Antibodies	NA
Peak calling parameters	NA
Data quality	NA
Software	NA

Flow Cytometry

Plots

Confirm that:

☐ The axis labels state the marker and fluorochrome used (e.g. CD4-FITC).

☐ The axis scales are clearly visible. Include numbers along axes only for bottom left plot of group (a 'group' is an analysis of identical markers).

☐ All plots are contour plots with outliers or pseudocolor plots.

☐ A numerical value for number of cells or percentage (with statistics) is provided.

Methodology

Sample preparation	NA
Instrument	NA
Software	NA
Cell population abundance	NA
Gating strategy	NA

☐ Tick this box to confirm that a figure exemplifying the gating strategy is provided in the Supplementary Information.

Magnetic resonance imaging

Experimental design

Design type	NA
Design specifications	NA
Behavioral performance measures	NA
Imaging type(s)	NA
Field strength	NA
Sequence & imaging parameters	NA
Area of acquisition	NA

Diffusion MRI ☐ Used ☐ Not used

Preprocessing

Preprocessing software	NA
Normalization	NA
Normalization template	NA
Noise and artifact removal	NA
Volume censoring	NA

Statistical modeling & inference

Model type and settings	NA
Effect(s) tested	NA

Specify type of analysis: ☐ Whole brain ☐ ROI-based ☐ Both

Statistic type for inference
(See Eklund et al. 2016)

NA

Correction

NA

Models & analysis

n/a	Involved in the study
☐	☐ Functional and/or effective connectivity
☐	☐ Graph analysis
☐	☐ Multivariate modeling or predictive analysis

Functional and/or effective connectivity

NA

Graph analysis

NA

Multivariate modeling and predictive analysis

NA

This checklist template is licensed under a Creative Commons Attribution 4.0 International License, which permits use, sharing, adaptation, distribution and reproduction in any medium or format, as long as you give appropriate credit to the original author(s) and the source, provide a link to the Creative Commons license, and indicate if changes were made. The images or other third party material in this article are included in the article's Creative Commons license, unless indicated otherwise in a credit line to the material. If material is not included in the article's Creative Commons license and your intended use is not permitted by statutory regulation or exceeds the permitted use, you will need to obtain permission directly from the copyright holder. To view a copy of this license, visit http://creativecommons.org/licenses/by/4.0/
